# Important Considerations for the Institutional Review Board When Granting Health Insurance Portability and Accountability Act Authorization Waivers

**DOI:** 10.31486/toj.19.0083

**Published:** 2020

**Authors:** Kelsey Williams, Paul Colomb

**Affiliations:** Department of Compliance and Privacy, Ochsner Clinic Foundation, New Orleans, LA

**Keywords:** *Ethics committees–research*, *Health Insurance Portability and Accountability Act*, *privacy*, *research*

## Abstract

**Background:** Privacy is recognized as a basic human right in the United States and has been identified as a core principle of ethics in clinical research. However, changes in the regulations, changes in how research is conducted, and the availability of health data stored in electronic health record systems all pose risks to individuals’ privacy.

**Methods:** The Health Insurance Portability and Accountability Act (HIPAA) Privacy Rule addresses the use and disclosure of individuals’ health information and sets standards for privacy rights so that individuals can understand and control how their health information is used. However, despite the significant increase in the complexity of the data privacy landscape, the HIPAA Privacy Rule has been largely unchanged since its enactment in 1996.

**Results:** Generally, healthcare entities may not use or disclose protected health information (PHI) for research without written authorization from each subject permitting that use or disclosure. However, the HIPAA Privacy Rule allows an institutional review board (IRB) to waive the need for such authorization if documentation is provided that the use or disclosure of PHI presents “no more than a minimal risk to the privacy” of the subjects. Because IRBs were one of the only bodies allowed to waive the need for authorizations in the research context, they essentially served as the gatekeepers of privacy for human subjects. However, this situation changed with the 2018 revisions to 45 CFR §46—known as the Common Rule—that added new categories of exempt research. Under the new regulations, research administrative staff may review a submitted research study and determine that it is exempt without the IRB ever being involved and with no independent review of privacy considerations. This change lessens privacy protections for research subjects. Therefore, IRBs must be mindful of the relevant HIPAA guidance and carefully consider all facts and circumstances available when granting approvals of HIPAA authorization waiver requirements, especially in the content of exempt research, so that the IRB is confident that reasonable safeguards to protect patient privacy have been maintained. Research institutions should amend their processes to ensure that the appropriate level of privacy review is given to all studies, even those that are exempt.

**Conclusion:** Few concrete rules are applicable in the research context that ensure compliance with the HIPAA Privacy Rule. Ultimately, more definitive regulatory guidance integrating HIPAA and the revised Common Rule should be promulgated.

## INTRODUCTION

Human subjects research drives medical advancements. This research is instrumental in the identification of the causes of certain diseases and the development of treatments for these diseases. However, the importance of conducting this research must always be balanced with the responsibility of protecting the human subjects who participate in the research. The regulations governing human subjects research are principally aimed at protecting research subjects and were developed, in part, in response to unethical research activities. The development of these regulations began with a focus on informed consent.

Many long-standing principles for conducting human subjects research originated with the Nuremberg Code, released in response to the “gruesome atrocities” committed in the pursuit of “Nazi medicine.”^1^ The Code, issued in 1947, consists of 10 principles centered on the tenets of voluntariness and the doctrine of informed consent. In 1974, the National Research Act, enacted by the 93rd United States Congress, created the National Commission for the Protection of Human Subjects of Biomedical and Behavioral Research.^2^ In 1979, that Commission released the Belmont Report which, like the Nuremberg Code, stresses the importance of informed consent. The Belmont Report sets forth 3 basic ethical principles: respect for persons, beneficence, and justice.^3^ Each of these principles contains the tenet of voluntary informed consent. The Federal Policy for the Protection of Human Subjects, promulgated by the US Department of Health and Human Services (HHS) and codified at 45 CFR §46, was adopted in 1991 by 15 federal departments and agencies. Subpart A of this regulation, generally known as the Common Rule, outlines the basic provisions for institutional review boards (IRBs), informed consent, and assurances of compliance with the policy.^4,5^

Because of these regulations and policies, the concept of informed consent is well developed. However, the nature of clinical research continues to change. Advancements in technology, including the development of electronic health record systems, enable the storage of large amounts of health data. These data are valuable to researchers because they provide vast research opportunities, but stored data present real risks to individuals’ privacy.

## A FUNDAMENTAL NEED FOR PRIVACY

Privacy is recognized as a basic human right in the United States and has been identified as a core principle of ethics in clinical research.^6^ Health data privacy protections were enacted in 1996 when the Health Insurance Portability and Accountability Act (HIPAA) was passed. HIPAA amended the Internal Revenue Code of 1986 with provisions to simplify the administration of health insurance, to enable the responsible flow of medical information to advance patient care, and to promote good clinical practice.^7^ Sections 261 through 264 of HIPAA were established, in part, with the purpose of improving “the efficiency and effectiveness of the health care system by encouraging the development of a health information system through the establishment of standards and requirements for the electronic transmission of certain health information.”^7^ The Standards for Privacy of Individually Identifiable Health Information, the HIPAA Privacy Rule (45 CFR §160 and Subparts A and E of §164), was promulgated under that stated purpose.^8^ The HIPAA Privacy Rule addresses the use and disclosure of individuals’ health information by certain organizations and sets standards for individuals' privacy rights so that they can understand and control how their health information is used.^9^

Despite the tremendous increase in the complexity of the data privacy landscape, both within the healthcare industry and beyond, the HIPAA Privacy Rule has been largely unchanged since its enactment. Thus, the privacy burden has generally fallen on organizations to analyze and interpret the aging rules and apply them to novel concepts, such as data collection from wearable health devices, a situation almost certainly not foreseen by the rule's original authors.

## CURRENT FRAMEWORK FOR PRIVACY PROTECTION IN CLINICAL RESEARCH

HIPAA promotes the advancement of good medicine by enabling the sharing of medical information with providers for continuity of care, and the HIPAA Privacy Rule establishes the conditions under which protected health information (PHI) may be used or disclosed for research purposes.^10^ Generally, healthcare entities may not use or disclose PHI in the context of research without written authorization from each subject allowing for that use or disclosure. However, the HIPAA Privacy Rule allows an IRB, a committee charged with reviewing and approving human subjects research, to waive the need for an authorization. The IRB may only waive the need for an authorization if documentation is provided that shows the use or disclosure of PHI presents “no more than a minimal risk to the privacy” of the subjects.^11^ An adequate plan to protect the identifying information contained in PHI from improper use or disclosure must be in place, as well as an adequate plan to destroy that information at the earliest opportunity consistent with the conduct of the research. Additionally, adequate written assurances must be provided that the PHI will not be reused or disclosed to any other person or entity, except as required by law, for authorized oversight of the research study or for other research for which the use or disclosure of PHI would be permitted by law.

Because IRBs were one of the only bodies allowed to waive the need for authorizations in the research context, they essentially served as the gatekeepers of privacy for human subjects. However, this situation changed with the 2018 revisions to the Common Rule.^12^

Under the Common Rule, IRBs must review and have authority to approve, require modifications in, or disapprove “all research involving human subjects conducted, supported, or otherwise subject to regulation” by any of the federal agencies that promulgated or have adopted the Common Rule.^12^ However, certain categories of research are exempt from the Common Rule requirements that the researcher obtain IRB review and approval of the research and informed consent of the research subject. The 2018 revisions added new categories of research that are exempt, including secondary research regulated under the HIPAA Privacy Rule. Secondary research is the reuse of identifiable information and identifiable biospecimens originally collected for some other research or healthcare activity, such as research that uses PHI from an existing databank.^13^ HHS made this change because the agency considers secondary research to be associated with sufficiently low human subject research risks, and the important risk of privacy should be governed by the HIPAA Privacy Rule instead of the Common Rule.^14^ However, under the revised Common Rule, research administrative staff may review a submitted research study and determine that it is exempt without the IRB ever being involved and without any independent review of privacy considerations. This situation potentially lessens privacy protections for research subjects, placing the burden for privacy protection on IRBs and research institutions.

Therefore, as in almost every decision a healthcare organization makes with respect to handling of PHI, IRBs must be mindful of the relevant HIPAA guidance and must carefully consider all facts and circumstances available when granting approvals of HIPAA authorization waiver requirements, especially in the context of exempt research, so that the IRB is confident that reasonable safeguards to protect patient privacy have been maintained. Research institutions should amend their processes to ensure that the appropriate level of privacy review is given to all studies, even those that are exempt.

## CONCLUSION

The HIPAA Privacy Rule is a set of guidelines that requires reasonable safeguards to protect patient privacy to be applied in any situation involving the use or disclosure of PHI, but few concrete rules apply to the research context that ensure compliance. Ultimately, more definitive regulatory guidance integrating HIPAA and the revised Common Rule should be promulgated, as the revised Common Rule itself promotes.

## Notes

This article meets the Accreditation Council for Graduate Medical Education and the American Board of Medical Specialties Maintenance of Certification competencies for Patient Care, Medical Knowledge, and Systems-Based Practice.
